# Advantages of Intraoperative Neuromonitoring Over Direct Visualization of the Recurrent Laryngeal Nerve During Thyroidectomy

**DOI:** 10.7759/cureus.43869

**Published:** 2023-08-21

**Authors:** Mauricio Gutierrez-Alvarez, Jorge Alejandro Torres-Ríos, Michelle Torreblanca-Olascoaga, Ana Priscila Campollo-Lopez, Fernando Barbosa-Villarreal, Alejandra Judith Padilla-Flores, Jorge Leal, Cielo Silva, Jorge Alberto Robles-Aviña

**Affiliations:** 1 General Surgery, Medica Sur, Mexico City, MEX; 2 Member of the Mexican Faculty of Medicine, Universidad La Salle Mexico, Mexico City, MEX; 3 Neurosurgery, Insituto Nacional de Neurología y Neurocirugía Manuel Velasco Suárez, Mexico City, MEX; 4 Anesthesiology, Medica Sur, Mexico City, MEX; 5 Plastic Surgery, Hospital Central Sur de Alta Especialidad, Pemex, Mexico City, MEX; 6 Plastic Surgery, Hospital central sur de alta especialidad de Pemex, Mexico City, MEX; 7 Surgery, Medica Sur, Mexico City, MEX; 8 Surgical Oncology, Medica Sur, Mexico City, MEX; 9 Surgical Oncology, Hospital General Dr. Manuel Gea González, Mexico City, MEX

**Keywords:** intraoperative neuromonitoring, recurrent laryngeal nerve, nerve injury, thyroidectomy, neuromonitoring

## Abstract

Background: The well-recognized risk of injury to the recurrent laryngeal nerve (RLN) during thyroidectomy has instigated various preventive measures. One such measure involves directly visualizing the RLN, but this is not always feasible in practice. A more recent approach involves using intraoperative neuromonitoring to identify and preserve the RLN. This study aims to evaluate the effectiveness of intraoperative neuromonitoring compared to single visualization of the RLN in averting nerve injury.

Methods: We conducted a retrospective, observational, and descriptive study on a cohort of 218 patients. A Chi-square test was employed to determine the influence of intraoperative neuromonitoring on the incidence of nerve injury, with P < 0.05 considered statistically significant. We used Jamovi software version 2.3.18 to analyze the data.

Results: Of the 218 patients, intraoperative neuromonitoring was used in 150 (68.8%) cases; none of which resulted in nerve injury. Conversely, 68 (31.2%) patients underwent surgery without the use of neuromonitoring, with two (2.9%) patients in this group experiencing nerve injury (p=0.037). In comparison, the risk of nerve injury was 0% in the group monitored intraoperatively and 2.94% in the group that did not undergo intraoperatively neuromonitoring. Further, the relative risk of complications was 0.66% in patients operated with neuromonitoring, while it was 5.88% in the group operated without neuromonitoring, thus demonstrating a clinically significant protective against vasculonervous complications.

Conclusion: The results advocate for the use of intraoperative neuromonitoring, whenever available, as it is a safe method for significantly decreasing the incidence of RLN injury during thyroidectomy compared with only visualization.

## Introduction

Thyroidectomy is a surgical technique that consists in resection of the thyroid gland due to an incision in the neck, this is used to treat benign and malignant lesions of the thyroid. There have been reports of thyroid resections since ancient times, however, modern thyroidectomy was described by Professor of Surgery in Bern, Switzerland Theodore Kocher, who is also a winner of the 1909 Nobel Prize in Medicine for his research on thyroid surgery.

During thyroid surgeries, it is important to avoid injury to the recurrent laryngeal nerve (RLN) [1] due to the complications it can generate, such as dyspnea, airway obstruction or voice changes [1-3]. A temporary or permanent lesion of RLNs is the most serious complication during thyroid surgery [4,5]. The incidence of this event ranges from 2% to 13% [6], with temporary lesions occurring in 2% to 11% of cases and permanent lesions occurring in 0.6% to 2.3% of cases [4,5].

Visual nerve identification has been proposed with the aim of avoiding injuries; to date, this measure has caused a decrease in the incidence of injuries to the RLN [6,7]. This practice is not always possible; thyroidectomies due to invasive neoplasms [1,2,6], thyrotoxicosis [6], Graves' disease or anatomic variations are considered high risk due to the difficulty for nerve visualization [1,6]. Another significant factor in the presentation of lesions is the surgeon’s level of expertise [2]. Intraoperative neuromonitoring (IONM) began to be utilized at the end of the 1960s and has steadily gained popularity in recent years [8]. Currently, the advantages of IONM over nerve visualization have been demonstrated, particularly in the high-risk groups of patients [1]. Since there are still studies and meta-analyses that have not found significant differences [1,6,9,10] in addition to the higher surgical cost [4,11,12], there is still no adequate consensus to standardize its routine use. We intend to compare the use of IONM against only direct visualization of the RLN in a Mexican population in a hospital located in the south of Mexico City.

## Materials and methods

**Figure 1:**
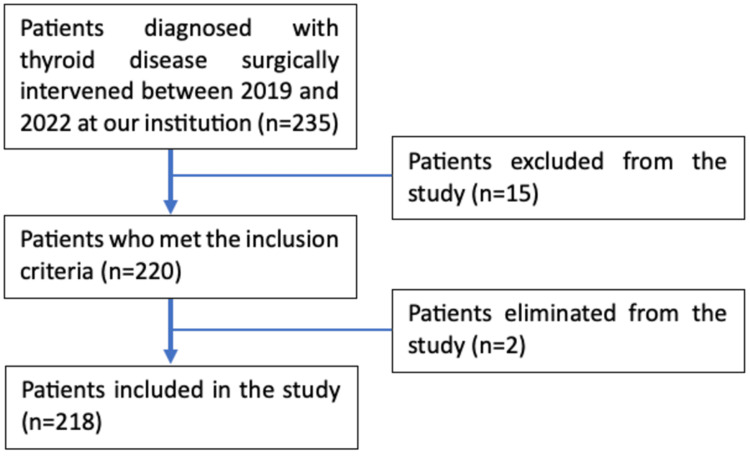
Algorithm for patient selection.

This was a retrospective, observational, and descriptive cohort study. The population of interest comprised all patients with a diagnosis of thyroid pathology who underwent treatment in the surgery department of our institution between the years 2019 and 2022. The inclusion criteria employed in this study encompassed patients who had undergone either partial or total surgical resection of the thyroid gland with postoperative follow-up and complete medical records between 2019 and 2022. These patients were required to possess a histological diagnosis, have received immediate postoperative clinical follow-up, and possess complete clinical records. Between January 2019 and December 2022, a total of 235 patients who underwent partial or total resection of the thyroid gland were included in the study using a non-probabilistic sampling method based on convenience. Among these patients, only 220 met the inclusion criteria. Two patients were excluded from the study as they had a history of previous surgical treatment. Ultimately, 218 patients participated in the study (Figure 1).

The primary objective of this study is to determine the effectiveness of IONM during thyroidectomy in reducing the risk of RLN injury. The secondary objectives are to describe the characteristics of the study population, evaluate the impact of IONM on intraoperative bleeding and duration of operative time, and provide a description of complications related to the procedure.

All patients were treated at the same institution by 20 surgeons from different specialties, including two general surgeons, 12 oncologic surgeons, one head and neck surgeon, five endocrine surgeons and two otolaryngologists. The experience among surgeons was diverse. In all cases, Nerve Integrity Monitoring System (NIM)-Response 3.0® (Medtronic Xomed®) was utilized.

Regarding statistical analysis, frequency analysis was performed for nominal variables. Kolmogorov-Smirnov normality tests were applied, and then mean and deviation were utilized to describe normally distributed numerical variables and median and range to describe non-normally distributed numerical variables. The Chi-squared test was utilized to evaluate the impact of neuromonitoring on the frequency of nerve and vascular injury. The Student's t-test was utilized to evaluate the impact of IONM on the duration of operative time and days of hospital stay. The results were declared significant for p-values <0.05. Statistical analysis was performed with the Jamovi software version 2.3.18. A search for articles was carried out in PubMed, the Cochrane Library, Google Scholar and a synthesis of current knowledge.

## Results

The study included 218 patients, of whom 179 (82.1%) were female, and 39 (17.9%) were male, resulting in a female-to-male ratio of approximately 4.5:1. Among these patients, 137 (62.8%) patients had an oncologic diagnosis. Regarding histopathological diagnosis, 77 (35.3%) had benign pathologies, such as multinodular goiter, thyroid adenoma, Hashimoto's thyroiditis, and thyroid nodule; 131 (60%) had papillary thyroid carcinoma, eight (3.7%) had follicular thyroid carcinoma, one (0.5%) had medullary thyroid cancer and one (0.5%) patient had anaplastic thyroid carcinoma.

**Figure 2:**
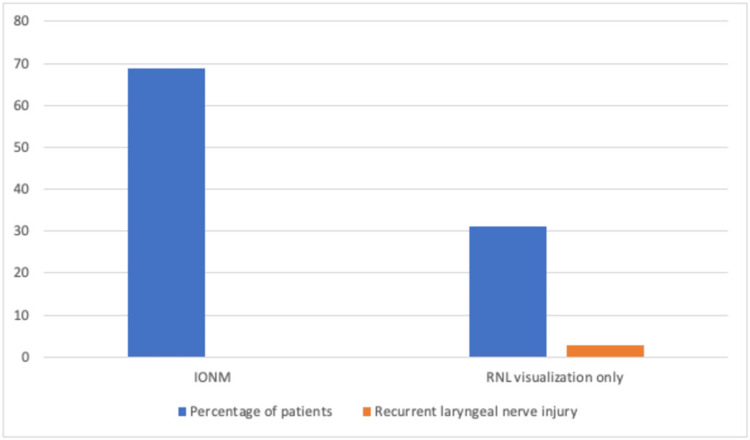
Comparison of patients who underwent thyroidectomy with the use of intraoperative neuromonitoring versus direct visualization only and the risk of recurrent laryngeal nerve injury associated with each of the procedures. n = 218 RLN: recurrent laryngeal nerve, IONM: Intraoperative neuromonitoring

IONM was used in 150 (68.8%) patients, of which none presented nerve damage. However, 68 (31.2%) patients were operated on without using neuromonitoring, and only two (2.9%) patients presented nerve injury (p = 0.037) (Figure 2). Therefore, the risk of nerve injury in the group with neuromonitoring was 0%, while in the group without neuromonitoring, it was 2.94%. It was not possible to calculate the relative risk because no patient presented a nerve injury in the group operated on with IONM.

Regarding vascular injury, in the group of patients with IONM, only one (0.6%) patient presented vascular injury, while three (4.4%) patients presented this complication in the group operated on without neuromonitoring (p = 0.060). In addition, the risk of vascular injury in the group operated on with IONM was 0.66%, while in the group operated on without neuromonitoring, it was 4.41%. The relative risk of developing vascular injury using IONM was 0.15 with a 95% confidence interval (0.01-1.43), showing a protective factor of neuromonitoring for the development of vascular injury but without clinical significance. 

Therefore, regarding the frequency of nerve injury, a significant difference was found in favor of the use of IONM. Moreover, when assessing the risk, a clinically significant difference was found in the relative risk of developing complications, indicating that IONM acts as a protective factor.

**Figure 3:**
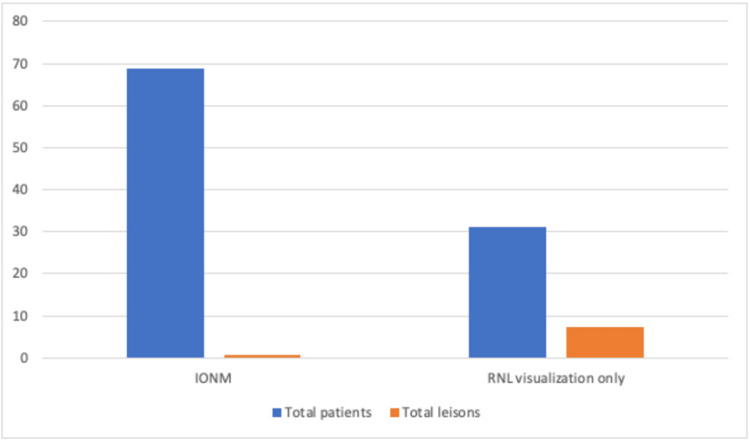
Total nerve and vascular lesions in patients with intraoperative neuromonitoring and in patients without neuromonitoring during thyroidectomy. n = 218 RLN: recurrent laryngeal nerve, IONM: Intraoperative neuromonitoring

Out of the 218 patients enrolled in the study, only six (2.7%) presented complications; two were nerve complications (both of them were due to transection), and four were vascular lesions. When considering all complications, only one patient (0.6%) was in the group of patients operated on with IONM, and five (7.3%) were in the group without IONM (p = 0.061) (Figure 3). In addition, the relative risk of complications was 0.66% in the group operated on with IONM, while the group operated on without neuromonitoring had a risk of 5.88%. The relative risk of developing complications was 0.09 (the risk of developing a complication using neuromonitoring is 9% compared to procedures without neuromonitoring) with a 95% confidence interval (0.01-0.76), showing a protective factor for the development of vasculonervous complications with clinical significance.

The median operative time across the entire study population was 105 minutes, with a range of 43 to 415 minutes. In the group of patients operated on with IONM, the median operative time was 100 minutes, with a range of 43 to 360 minutes. In the group of patients without IONM, the median operative time was 115 minutes, with a range of 50 to 415 minutes, although these differences were not statistically significant (p = 0.154). In the group of patients operated on by endocrine surgeons, the mean operative time was shorter, followed by the group operated on by oncologic surgeons and head and neck surgeons. The comprehensive findings are presented in Table 1.

**Table 1:** Surgical time by specialty ST: surgical time, ONC: oncology, END: endocrinologists, H&N: head and neck, GS: general surgery.

	ST OTR	ST ONC	ST END	ST H&N	ST GS
N	6	73	126	9	3
Mean	200	127	110	154	140
Estandar deviation	95.7	62.2	40.6	51.6	18.0

Furthermore, the median length of hospital stay was one day, ranging from one to 13 days. The median length of hospital stays in the group operated on with IONM was 1.52 days (± 1.11 days), while in the group of patients operated on without IONM, it was 2.04 days (± 1.71 days). Upon statistical analysis, a statistically significant difference was observed between the two groups in the days of hospital stay (p = 0.010).

## Discussion

IONM in thyroid surgery is performed through an electromyography signal of vocal muscle movement to reflect the function of the RLN [5,8]. This group has two generations of neuromonitoring: continuous and non-continuous monitoring. The latter is the most prevalent and is utilized by the surgeons in our study. Continuous neuromonitoring is a much more recent and modern technique; it sends information in real-time [5]. Surveys suggest that in the United States and Europe, more than 90% of endocrine and head and neck surgeons use IONM routinely [8]. In our study, the percentage was lower, at 68.8% of cases.

Taking into account that the IONM offers immediate audible and visual information and that injuries to the RLN are produced by complete nerve section, pinching, stretching, electrothermal injury, entrapment or ischemia during the surgical procedure [1,2,5,6], the IONM assists in guiding us towards the tissue that requires additional care and sectioning without complications [2]. According to certain surveys conducted by endocrine surgeons with extensive experience, it has been observed that between 6.8% and 11.5% of thyroidectomies, the identification of nerve structures is not feasible, thereby increasing the likelihood of nerve injury in this group of surgeons [4]. Consequently, for novice surgeons, the risk may be even higher. In our study, the most severe injury to the RLN was observed in inexperienced surgeons, i.e., general surgeons. As a result of the complete injury and total collapse of the vocal cords, a tracheostomy was necessary to maintain airway permeability in the future, an event which could be preventable with the use of IONM as in the IONM group none presented nerve damage. In addition to nerve injury, another complication found in our study was vascular injury, present in three patients (4.4%) without the use of IONM and one patient (0.6%) with the use of IONM. There was no significant statistical difference between the two groups (p = 0.060) and this was simply an observation from the available data. Clinically, RLN lesions present as dysphonia, dysphagia, and fluid aspiration, which can lead to airway obstruction, necessitating emergency tracheostomies during the early postoperative period [4].

In our study, a significant statistical difference was observed in favor of the use of IONM to avoid lesions to the RLN. Lesions were present in 2.9% of the patients who underwent surgery without IONM, while in the group operated on with IONM, there were no nerve lesions (p = 0.037). Our results are similar to recent studies such as the one performed by Martucci C et al., who have also seen the benefit of IONM over nerve visualization alone, especially in high-risk patients where visualization is difficult [1]. Another study that supports our results is one of the most recent and largest meta-analyses conducted by Bai and Chen, where 34 articles were included in the systematic review. It was found that IONM does have positive benefits in terms of reduction of partial and total lesions of the RLN compared to visual identification, presenting a significant decrease in the total lesion of the RLN (RR = 0. 68; 95% CI: 0.55 to 0.83; p = 0.0002), transient injury (RR = 0.71; 95% CI: 0.57 to 0.88; p = 0.0017) and permanent injury (RR = -0.0026; 95% CI: -0.0039 to -0.0012; p = 0.0003) by IONM [5].

Another advantage of the utilization of IONM is the reduction of surgical time, owing to the earlier identification of the RLN [3,6]. In the study, a significant difference in the reduction of surgical time was not established (p = 0.154), although the average time in the IONM group was 100 minutes compared to 110 minutes in the group that did not use IONM. A larger sample size would probably make a clinical difference. Despite this limitation and the results in our study, there are authors such as Princi et al. who report a reduction in the duration of surgical time from 128.1±39.3 minutes in the control group with only nerve visualization to 106.3±38.7 minutes with the use of IONM (p=0.014) [13]. Fei et al. observed a reduction in the time taken to identify RLN in favor of the group where IONM was utilized in the complete endoscopic radical resection of thyroid cancer [14]. In addition, a statistically significant reduction in hospital stay time was observed with a median time of 1.52 days (± 1.11 days) in the group operated on with IONM, while in the group of patients operated on without IONM, it was 2.04 days (± 1.71 days) (p = 0.010). The median length of hospital stay was one day in a range of one to 13 days, with 13 days for a patient operated on without neuromonitoring and presenting severe complications.

As mentioned by Pietro Giorgio et al., the utilization of IONM can also provide surgeons with greater assurance of the integrity of RLN at the conclusion of a surgical procedure. Many times, injuries are not evident until early postoperatively, and some studies have even shown that, in most cases, surgeons were unaware of the injury during the procedure [2]. It is estimated that only 15% of the lesions are detected physically [4], while with the use of IONM, they can be identified close to 100% in the trans-surgery [4,14], assessing the integrity of the RLN at the end of the procedure [2,15]. In our instance, the lesions were not discernible during the intraoperative period, but they became apparent during the postoperative period. This enabled IONM to assist in the intraoperative identification of lesions to the RLN and exercise greater caution in postoperative management and surveillance.

However, despite the results obtained in favor of the use of IONM, the usefulness of routine IONM is still in question since some studies and meta-analyses, such as the one performed by Davey et al. conclude that there are no significant differences between the use of IONM and direct visualization [8]. Yarbrough et al., in their study with 52 patients, obtained results against the use of IONM since they did not observe a significant advantage [9]. In this particular instance, it is worth noting that the sample size is limited. However, a recent study conducted by Goretzki et al. presents compelling evidence opposing the widespread utilization of IONM. The study, which included a total of 1,333 patients, did not yield statistically significant differences in outcomes [10]. Similar to the aforementioned study, several others continue to demonstrate non-significant variances and oppose the routine utilization of IONM [1,2,15,16]. Martucci et al. have concluded that there is no significant benefit in favor of IONM compared to direct visualization of the RLN in terms of lesion reduction in the pediatric population, who have smaller surgical spaces compared to adults, making them more prone to RLN lesions [1]. However, it is important to note that IONM is an innocuous study and has no associated complications [2].

One of the main disadvantages of the use of IONM is its cost [4,11], which can increase by 5% to 7% [8] and also lead to longer patient preparation time [2]. As a result, standardizing the use of IONM in surgeries that are not considered high-risk is currently challenging from a cost-benefit perspective. However, according to Chan et al., it could still be recommended for high-risk surgeries [17]. Based on the results of our study, we are in favor of the use of IONM due to the statistically significant reduction of injuries to the RLN. In private medicine, an area where medico-legal cases are more frequent, IONM may be useful as medico-legal protection [2,5]. Some studies have already shown that IONM associated with direct visualization of the RLN reduces the injury rate considerably [2,4] without lengthening surgical time [1].

The main limitation of our study is that it was not performed in a randomized fashion, which may lead to biases. On the other hand, all surgeons from different specialties were included, which generates a slight bias in terms of experience since it can be seen which general surgeons had a higher injury rate. A study with a larger sample may yield more reproducible results. The rising costs make routine IONM not cost-effective in countries such as ours.

The most widely used IONM method is intermittent; however, technological advances have opened the doors to continuous IONM, which could have greater advantages over direct visualization and intermittent IONM of the RLN. Clinical trials that compare continuous and intermittent IONM represent a promising area in the field of surgery.

## Conclusions

Despite the evidence in favor of the use of IONM during thyroidectomy, there are still studies opposing the utilization of IONM. In our study, a significant statistical difference was observed in favor of the use of IONM to avoid lesions to the RLN injury. Lesions were present in 2.9% of the patients who underwent surgery without IONM, while in the group operated on with IONM, there were no nerve lesions. However, we recommend the use of IONM whenever it is available because it is a safe method and there are studies like ours that have found statistically significant differences in terms of reducing the rate of RLN injury thus avoiding postoperative complications. On the other hand, the surgical time has no significant difference in both groups; however, there is a significant difference in the length of hospital stay. The main limitation of our study is that it was not performed in a randomized fashion, which may lead to biases.
